# Pseudo-vagal Responses Elicited by Cryoballoon Ablation

**DOI:** 10.19102/icrm.2023.14123

**Published:** 2023-12-15

**Authors:** Daniel Sohinki, Vinay Mehta, Jeffrey Ardell, Stavros Stavrakis, Sunny S. Po, Ali Yousif

**Affiliations:** 1 Division of Cardiology, Medical College of Georgia, Augusta, GA, USA; 2 Cardiac Arrhythmia Center, David Geffen School of Medicine, University of California, Los Angeles, CA, USA; 3 Section of Cardiovascular Diseases and Heart Rhythm Institute, University of Oklahoma Health Sciences Center, Oklahoma City, OK, USA; 4 Department of Cardiology, Baylor Scott and White Health, Prosper, TX, USA

**Keywords:** Atrial fibrillation, autonomic nervous system, cryoballoon, vagal

## Abstract

Pulmonary vein isolation via cryoballoon (CB) ablation is the cornerstone ablation strategy for the treatment of atrial fibrillation (AF). Acute intraprocedural hypotensive and/or bradycardic responses have been reported in patients undergoing CB ablation for AF. However, it remains unclear as to whether these are due to a true vagal response (VR), which can be used to predict long-term outcomes of CB ablation. We analyzed 139 freezes across 17 patients who received CB ablation for paroxysmal AF, measuring vital signs and freeze characteristics. Only one freeze was associated with both hypotension and bradycardia, constituting a true VR. Several freezes were associated with hypotension only that did not respond to atropine administration, suggesting that these responses are not associated with a VR. Hypotensive responses were significantly associated with ice bubble bursts during CB deflation. Unlike the true VR reported in patients undergoing conscious sedation, the presence of acute hypotension shortly after CB deflation cannot be used as a predictor for long-term ablation outcomes.

## Introduction

Pulmonary vein isolation (PVI) is the cornerstone ablation strategy for the treatment of atrial fibrillation (AF).^1^ PVI accomplished by cryoballoon (CB)-based catheter ablation is comparable to radiofrequency (RF)-based catheter ablation with respect to efficacy and safety in patients with paroxysmal AF.^2,3^

Acute intraprocedural hypotensive and/or bradycardic responses have been reported in patients undergoing CB or RF ablation for AF.^4,5^ These acute changes consisting of a drop in blood pressure (BP), sinus bradycardia, asystole, or atrioventricular (AV) block are collectively being attributed to vagally mediated responses by the delivery of either cryo or RF energy.^5,6^ In one study, nearly half of the patients undergoing CB ablation using the latest CB technology (Arctic Front Advance™; Medtronic Inc., Minneapolis, MN, USA) experienced a “vagal response” (VR).^7^ This VR is usually temporary and often does not require any pacing or atropine administration.

Some studies showed that the presence of a VR during CB application predicts intrinsic cardiac autonomic nervous system (ANS) modulation and/or denervation via direct effects on ganglionated plexi (GP).^5,6^ The benefits of ANS modulation with CB ablation on AF outcomes have been mixed. Oswald et al. showed that, using measurements of heart rate variability as an indirect measure of ANS activity, the long-term outcome of AF-free survival was similar between patients with and those without ANS modification.^6^ Contrarily, Aytemir et al. demonstrated that intraprocedural VR was associated with lower AF recurrences in patients undergoing CB ablation for drug-refractory symptomatic AF.^7^

Current published data suggest that the incidence of “VR” during CB ablation for AF ranges from 35%–64%.^5–9^ Higher rates are seen with the second-generation CB.^7^ Recent studies have demonstrated VR reversal with intravenous atropine administration, while others reported no effect on BP or heart rate.^9,10^ Therefore, it remains unclear whether the acute hypotensive and/or bradycardic changes during CB ablation are due to a VR. Given the possible association of GP ablation and VR on AF outcomes, it is important to confirm whether these responses are in fact VRs. Physicians may falsely assume that a “VR” during cryoablation is indicative of GP ablation and may feel the need to perform further ablation if this response is not seen. In addition, unnecessary interventions or procedural delays may be instituted in response to these hemodynamic changes if they are not expected by the electrophysiology or anesthesia team. Finally, it is important to clarify the underlying hemodynamic changes that occur during CB ablation in order for physicians to have a better understanding of the impact of ablation on cardiac ANS function in general. We aimed to investigate alternative factors and triggers that contribute to BP and heart rate changes during CB applications.

## Methods

### Electrophysiological study and ablation procedure

The current study was approved by the institutional review board and conducted in accordance with the principles of the Declaration of Helsinki. We enrolled 17 consecutive patients who presented to University of Oklahoma Health Science Center in sinus rhythm for first-time CB ablation to treat symptomatic paroxysmal AF. Anti-arrhythmic agents were held for at least five half-lives. Anticoagulation was held the night prior to the procedure. The day prior to the ablation procedure, all patients underwent preprocedural imaging that included computed tomography angiography and transesophageal echocardiography.

All ablation procedures were performed under general anesthesia, with volatile anesthetics such as sevoflurane or desflurane being the main agents used to maintain general anesthesia. Paralytic agents were used only during induction. Invasive arterial BP monitoring, oxygen saturation, and electrocardiograms were monitored continuously throughout the procedure. Multi-electrode catheters were inserted into the venous sheaths and advanced into the right atrial appendage and coronary sinus (CS). A transseptal puncture was performed with the utilization of intracardiac echocardiography (ICE) and fluoroscopy. The transseptal sheath SL1 was exchanged for a 12-French steerable transseptal sheath (FlexCath, CryoCath; Medtronic Inc.) over a stiff guidewire. A 28-mm CB and an inner circular catheter (Achieve Mapping Catheter; Medtronic Inc.) were used in all patients for PVI. The activated clotting time was between 300–350 s.

The balloon was guided toward each respective pulmonary vein (PV) ostium, then inflated, and an adequate seal was confirmed with fluoroscopy by injection of 50% diluted contrast through the CB catheter and with ICE by using color Doppler. A proximal seal technique was used to ensure a more antral freeze. PV potentials were monitored for recording the time to effect. Non-stacked 180-s freezes were delivered to each vein. If the time to effect was <60 s, a single 180-s application was applied to that PV. PVI was performed in the following order: left superior PV (LSPV), left inferior PV (LIPV), right inferior PV (RIPV), and lastly right superior PV (RSPV). Esophageal temperature was monitored in all cases. CB application was stopped prematurely for an esophageal temperature cutoff of 30°C. During RSPV CB freeze applications, pacing of the right phrenic nerve was performed to monitor phrenic nerve injury via direct palpation of right hemi-diaphragmatic excursions.

### Freeze and vital sign measurements

For each CB application, the total number of freezes, the duration of freeze, time to effect, and balloon and esophageal nadir temperatures were recorded. During each CB application, BP and heart rate were continuously monitored. If CS pacing was required for the visualization of PV signals to accurately record the time to effect, CS pacing was stopped before the end of the application to accurately reflect the sinus rate. During the thawing and balloon deflation phase, BP and heart rate were continuously monitored, and the lowest number was then recorded. Time to the lowest temperature was also recorded for each freeze application. ICE was used to visualize the balloon during each freeze application, balloon thawing, and balloon deflation post-thawing. In seven patients, 1 mg of atropine was administered intravenously prior to CB application. The heart rate and BP responses following atropine administration and CB application were recorded.

### Statistical analysis

Descriptive data, including patient demographics, number of freezes per vein, freeze duration, time to effect, balloon and esophageal temperature data, and time to the appearance of ice bubbles, are presented as mean ± standard deviation values. Analysis of variance (ANOVA) was used to compare these variables across veins. Where a difference was found, least squared means were used to make pairwise comparisons between veins. The Tukey–Kramer adjustment was used to account for multiple comparisons.

A multiple linear regression approach was used to compare the change in heart rate and systolic (SBP) and diastolic BP (DBP) between freezes where ice bubbles were seen and where ice bubbles were not seen. The change in BP or heart rate was calculated as the difference between BP or heart rate within 5 s before CB application was turned off and the minimum BP or heart rate for each freeze. ANOVA was used to compare freezes where atropine was administered to freezes where it was not. This model included a test for interaction between the presence of bubbles and the administration of atropine. Baseline BP (ie, BP recorded at the time cryoablation was turned off) was used as a covariate in the model to adjust for differences in the baseline BP measurement. Residual plots were created which validated the assumptions of linearity and equality of variance. Linear regression was also used to model the predictive ability of the lowest esophageal temperature with respect to the change in BP. The lowest esophageal temperature was also divided into quartiles, with BP changes in the first, second, and third quartiles all compared to that in the highest quartile.

ANOVA was used to compare the change in BP noted with ablation of each vein in cases where bubbles did or did not occur. The presence or absence of bubbles after ablation of each vein was used as a covariate in this model to assess for interaction between the presence of bubbles and each particular vein. Least squared means were used to make pairwise comparisons between each possible combination of veins (eg, LSPV vs. RSPV, LSPV vs. RIPV) where bubbles did versus where bubbles did not appear. The Tukey–Kramer adjustment was used to account for multiple pairwise comparisons. For all statistical analyses, a *P* value of <.05 was used to define statistical significance. Statistical analysis was performed using SAS version 9.4 (SAS Institute, Cary, NC, USA).

## Results

Data from a total of 17 patients were analyzed for the study. Baseline demographic data are given in **Table 1**. A total of 139 freezes were analyzed. Of note, only one CB application resulted in both hypotension and bradycardia, suggestive of a VR (sinus rate and BP dropped from 48 bpm and 102/48 mmHg to 34 bpm and 61/32 mmHg). The characteristics of all CB applications are presented in **Table 2**. As noted, the number of freezes per vein, mean freeze duration, time to appearance of ice bubbles, and coldest balloon temperature did not differ between veins **(Table 2)**. There was a significant difference in the time to effect or nadir esophageal temperature. On comparison of least squared means, the LIPV and RSPV differed significantly, with the freezes in the LIPV demonstrating a longer time to effect (70.9 ± 42.2 vs. 33.0 ± 24.4 s; *P* = .04) and a lower nadir esophageal temperature (32.3°C ± 4.2°C vs. 35.5°C ± 0.6°C; *P* = .005) as compared to the RSPV.

**Figure 1:**
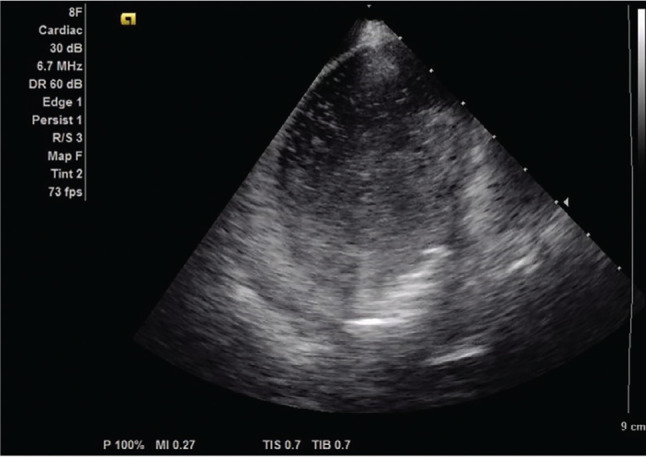
Appearance of a burst of ice bubbles after the cryoballoon warmed up and was deflated.

**Figure 2:**
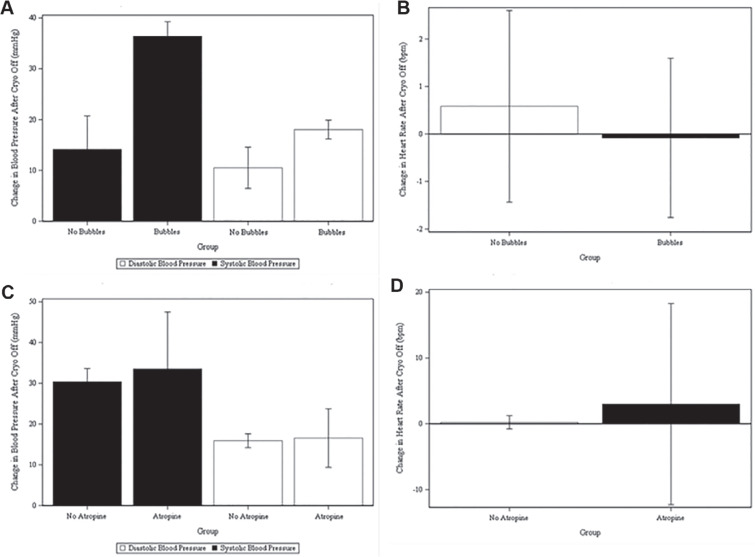
Blood pressure and sinus rate changes. **A:** Change in blood pressure after cryo off compared between groups. **B:** Change in heart rate after cryo off compared between groups. **C:** Change in blood pressure after cryo off compared between with and without atropine. **D:** Change in heart rate after cryo off compared between with and without atropine.

In the first 31 CB applications, we were not aware of the possible relationship between the appearance of a burst of ice bubbles and the hypotensive response. In the next 108 CB applications, we made every attempt to visualize the CB using ICE. The ICE image was adequate in 96 freezes to determine the presence or absence of ice bubbles. In 66 of 96 CB applications, ice bubbles were clearly visualized 27–36 s after the termination of the freeze **(Figure 1)**. Among CB applications with ice bubbles, 64 of 66 (97%) freezes recorded a significant drop in BP. In 24 of 30 (80%) freezes without ice bubbles, a drop in BP was also recorded. The mean SBP decrease was 36.42 ± 11.41 mmHg for freezes where ice bubbles appeared versus 14.15 ± 16.29 mmHg for freezes where ice bubbles did not appear **(Figure 2)**. This resulted in a 22.55- ± 2.85-mmHg greater drop in SBP for freezes where ice bubbles appeared after adjusting for baseline differences in SBP (95% confidence interval [CI], 16.88–28.22 mmHg; *P* < .0001). Similarly, the mean DBP decrease was 18.06 ± 7.06 mmHg for freezes where ice bubbles appeared versus 10.52 ± 10.26 mmHg for freezes where bubbles did not appear. This resulted in an 8.14- ± 1.81-mmHg greater drop in freezes where bubbles appeared after adjusting for baseline differences in DBP (95% CI, 4.54–11.75 mmHg; *P* < .0001) **(Figure 2)**. There was no significant difference in heart rate change between freezes where bubbles occurred and freezes where ice bubbles did not occur (0.58- ± 4.77-bpm increase for freezes without bubbles vs. 0.08- ± 6.55-bpm decrease in freezes with bubbles; 95% CI, −2.29 to 3.58; *P* = .66). The sinus rate was not affected by any of the 139 CB applications we investigated, regardless of the appearance of ice bubbles **(Figure 2)**.

We next used the ANOVA model to compare the change in SBP and DBP across veins in cases where ice bubbles did versus did not appear. In cases where ice bubbles did appear, the SBP drop did not differ between any two veins. There was no significant difference in DBP drop across veins. For freezes where ice bubbles did not appear, there was a greater drop in SBP during freezing of the LSPV as compared to the LIPV (*P* = .002) and the RIPV (*P* = .0041). There was also a trend toward a greater drop in SBP during freezing of the LSPV compared to the RSPV, though this result was not significant after adjusting for multiple comparisons (*P* = .2). There was no significant difference in SBP drop between any other pair of veins where ice bubbles did not appear.

Because of the prior suggestion that the hypotensive effect of CB applications was vagally mediated, we examined the effect of atropine administration on hemodynamics. There was no significant difference in SBP change (*P* = .27) or DBP change (*P* = .65) for freezes where atropine was administered versus freezes where it was not. The effect of atropine did not differ based on the presence or absence of bubbles (*P* for interaction = .38) **(Figure 2C)**.

**Figure 3:**
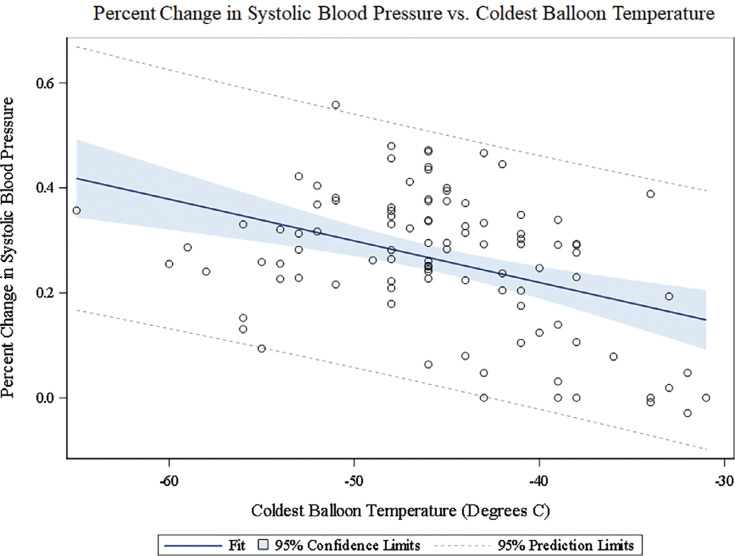
Correlation between the drop in systolic blood pressure and the coldest balloon temperature.

We next sought to examine the predictive ability of the coldest balloon temperature with respect to SBP and DBP changes. The ANOVA model demonstrated a modest negative correlation between the coldest balloon temperature and drop in SBP (*r* = 0.28; *P* = .01). That is to say, a lower coldest balloon temperature predicted a larger drop in SBP **(Figure 3)**. There was a trend toward a similar negative correlation between the coldest balloon temperature and DBP, but this was not statistically significant (*r* = 0.19; *P* = .06). **Table 3** gives a comparison between the first, second, and third quartiles of nadir balloon temperature against the highest quartile with respect to changes in SBP and DBP. As noted, the BP drop differed significantly between all quartiles as compared to the highest quartile, with the exception of DBP compared between the first and highest quartiles, where there was a non-significant trend (*P* = .13).

## Discussion

In the present study, we reported that the acute hypotensive response observed shortly after CB deflection was not accompanied by a corresponding bradycardic response. Atropine failed to affect the hypotensive response, indicating that it was not a true VR. However, the hypotensive response correlated well with the release of a burst of ice bubbles when the CB warmed up and was deflated. As the definition of a “VR” in prior studies included both bradycardia and hypotension, the incidence of “VR” may be overestimated. The conclusion that patients with “VR” elicited by CB ablation enjoy better long-term ablation outcomes may or may not hold true.

Human atria are richly innervated with the ANS, particularly the parasympathetic neural elements. Atrial autonomic innervation converges at several GP situated adjacent to the PV–atrial junction.^11^ The Oklahoma group demonstrated that the GP adjacent to the LSPV and RSPV are located within the range of the CB application. Isolation of the LSPV and RSPV eliminated the VR elicited by high-frequency stimulation delivered to the adjacent GP.^12^ Intuitively, one may expect to observe a VR during PVI. VRs were first described in RF ablation.^12^ The definition of a VR in the RF studies was sinus bradycardia (<40 bpm), asystole, AV block, or hypotension that occurred within a few seconds after the onset of RF application.^13^ The VR seen in RF studies occurred during RF application,^4,13^ while the VRs seen in CB ablation occurred during the thawing phase when the CB warmed up.^10,14^ However, the definition of a VR differed among CB ablation studies.^7,9,14^ The majority of studies did not specify which type of VR occurred but pooled all findings of bradycardia, sinus pause/arrest, hypotension, and AV block to report the incidence of VRs.^5–14^ This variability in definition accounts for the great difference seen in the reported incidence of VRs among studies.

Instead, we found that hypotension was the only hemodynamic change seen; it was transient, not accompanied by bradycardia or AV block. After we began to suspect a relationship between the appearance of ice bubbles and hypotension, we focused the ultrasound sector on the balloon–PV junction in the next 108 freezes; the balloon–PV junction was well visualized in 96 of 108 CB applications. Out of the 96 freezes, 66 freezes had a burst of ice bubbles seen on ICE on average 27–36 s after the termination of the freeze. There was a significantly larger drop in both SBP and DBP in the presence of ice bubbles. Intravenous atropine was administered prior to seven CB applications, which led to a mild increase in sinus rate without attenuating the hypotensive response. This finding suggests that hypotension occurring soon after CB deflation is unlikely to be vagally mediated. We further evaluated whether nadir balloon temperature affects the drop in BP by separating the balloon temperature into four quartiles. The first three quartiles had the coldest balloon temperature, which developed a statistically significant drop in BP in comparison to the fourth quartile that had the warmest balloon temperature. It is possible that a colder CB may release a larger volume of ice particles into the blood pool, thereby generating a larger hypotensive reflex. In freeze applications where ice bubbles did appear, there was no difference in the degree of BP drop among veins. The temporal relationship between appearances of ice bubbles and an acute drop in BP implies that the release of ice bubbles probably plays a role in acute hemodynamic effects. We hypothesize that this relationship is due to colder pulmonary venous blood in patients with noted ice bubble formation versus patients without ice bubble formation, presumably as a result of complete versus incomplete occlusion of the vein with the balloon. If cooling of the blood underlies the reflex mechanism seen, the response likely occurs on a spectrum based on the coldest temperature achieved in the blood. The sudden cooling of circulating blood may be related to the frontal headaches that patients complained about when CB ablation was performed with moderate sedation. While the exact mechanism of CB ablation–induced headaches is unknown, it is postulated to be similar to “brain freeze” or “ice cream” headaches that occur upon exposure to cold stimuli, such as an iced drink. Current theories for the “brain freeze” headaches hypothesize that the cool temperatures lead to cerebral vasodilation mediated through vessel nociceptor activation.^15^ Given the relationship between ice bubble appearance and the observed hypotensive response, a similar mechanism may be responsible for CB ablation–related frontal headaches.

It is known that cardiac pain is perceived through both cardiac visceral spinal afferent fibers and afferent vagal fibers after activation of the nociceptive receptors by tissue injury. Perception of pain can elicit the efferent vagal fibers through central processing.^16^ We hypothesize that nociception experienced by patients under conscious sedation may elicit stronger VRs that account for a much higher incidence of VR as compared to patients under general anesthesia who did not experience nociception.

In the present study, we only observed a single CB application that led to both bradycardia and hypotension, highly suggestive of a VR. This low incidence is consistent with findings of a prior study from our group^12^ but contrasts sharply with those of other studies reporting much higher incidence rates of “VR” after CB deflation in which ablation procedures were performed under moderate sedation.^5–14^ In our studies, all procedures were performed under general anesthesia using anesthetic gases (desflurane and sevoflurane), which do not have a significant effect on the cardiac ANS.

The occurrence of intraprocedural VR, seen as an indirect marker for cardiac ANS modulation, has been reported to be associated with a lower incidence of tachyarrhythmia recurrence after CB ablation for patients with paroxysmal^6,8^ or persistent AF.^17^ Interestingly, VRs were substantially more prevalent soon after isolating the first PV, particularly the LSPV. This observation was attributed to the atrial neural network that autonomic innervation to the sinoatrial node or AV node converges at the GP adjacent to the RSPV and RIPV.^18^ In the present study, LSPV was always the first vein to be isolated. The incidence of hypotension was not different among PVs, indicating different mechanisms from the VRs reported in sedated patients who can perceive pain. Given that the hypotensive response occurring in our anesthetized patients was not a true VR, hypotension alone occurring soon after CB deflation cannot be used as a predictor for better ablation outcomes.

### Study limitations

The current study has several important limitations. First, given our overall smaller sample size, our study has limited power to detect differences in response to cryoapplications, especially in cases where atropine was used. In addition, only a minority of patients had atropine administered to test the hypothesis that acute hemodynamic effects were related to a VR, lowering the power of our study to compare the atropine versus non-atropine freezes. Second, we only looked at patients with paroxysmal AF and did not include patients with persistent AF. Therefore, it is unclear whether this phenomenon would be present for patients with persistent AF who may have a greater degree of both structural and cardiac ANS remodeling. Third, no comparison was made between CB ablation patients and RF ablation patients, a potentially important comparator as VRs have been reported during RF ablation in stereotyped areas in the left atrium (eg, the ligament of Marshall). Fourth, we did not directly assess for GP denervation after ablation (eg, with high-frequency stimulation in the GP areas). Thus, while PVI was acutely successful and standard techniques were used, it cannot be definitively known whether the lack of a VR was because of incomplete autonomic denervation or because cryoablation truly does not elicit a VR. Fifth, while ICE was used in all cases to visualize the veins during freeze applications and to visualize the formation of ice bubbles, in some patients, evaluation for ice bubbles was limited, though we do have no reason to suspect that these patients differed systematically from patients in whom bubbles were easily visualized. Finally, we do not have long-term follow-up data on the patients in this study to assess ablation efficacy.

## Conclusion

The acute hypotensive response that occurred soon after CB deflation was not a true VR. The hypotensive response correlated well with the release of a burst of ice bubbles when the CB warmed up. Unlike the true VR reported in patients undergoing conscious sedation, the presence of acute hypotension shortly after CB deflation cannot be used as a predictor for long-term ablation outcomes.

## Figures and Tables

**Table 1:** Patient Demographic Data

Patients (n = 17)	
Age (years)	60.33 ± 12.53
LA size (cm)	3.9 ± 0.72
EF (%)	57.4 ± 4.45
Female sex	10 (67%)
Hypertension	10 (67%)
Type II DM	4 (27%)
CAD	3 (2%)
COPD	1 (7%)
CHF	1 (7%)
OSA	5(33%)

*Abbreviations:* CAD, coronary artery disease; CHF, congestive heart failure; COPD, chronic obstructive pulmonary disease; DM, diabetes mellitus; EF, ejection fraction; LA, left atrium; OSA, obstructive sleep apnea. Categorical data are listed as numbers.

**Table 2:** Cryolesion Characteristics Analyzed by Vein

Total Freezes = 139	LSPV	LIPV	RSPV	RIPV	*P* Value
Number of freezes (n)	1.74 ± 0.78	1.76 ± 0.75	1.76 ± 0.82	1.86 ± 0.89	.92
Freeze duration (s)	159.9 ± 31.5	159.8 ± 36.5	156.0 ± 32.0	146.4 ± 47.7	.46
Time to effect (s)	50.5 ± 33.4	70.9 ± 42.2	33.0 ± 24.4	35.5 ± 16.7	.04
Nadir esophageal temperature (°C)	33.6 ± 2.6	32.3 ± 4.2	35.5 ± 0.6	32.6 ± 3.8	.004
Time to bubble appearance (s)	27.9 ± 18.3	36.0 ± 25.8	27.6 ± 18.1	26.0 ± 18.5	.68
Coldest balloon temperature (°C)	−43.4 ± 19.9	−43.4 ± 5.8	−47.2 ± 8.4	−43.9 ± 7.4	.56

*Abbreviations:* ANOVA, analysis of variance; LIPV, left inferior pulmonary vein; LSPV, left superior pulmonary vein; RIPV, right inferior pulmonary vein; RSPV, right superior pulmonary vein. The ANOVA model revealed differences in time to effect, nadir esophageal temperature, and the coldest balloon temperature. During analysis of least squared means, time to effect was significantly longer in the LIPV as compared to the RSPV (*P* = .04), and nadir esophageal temperature was significantly lower in the LIPV as compared to the RSPV (*P* = .004).

**Table 3:** Comparison of Systolic and Diastolic Blood Pressure Changes by Quartile of Nadir Balloon Temperature

Quartile Comparison	SBP (mmHg)	*P* Value	95% CI (mmHg)	DBP (mmHg)	*P* Value	95% CI (mmHg)
First vs. fourth	11.35 ± 3.83	.02	1.37 to 21.34	4.37 ± 1.99	.13	−0.83 to 9.57
Second vs. fourth	19.02 ± 5.04	.001	5.88 to 31.17	7.72 ± 2.86	.04	0.27 to 15.17
Third vs. fourth	15.2 ± 3.77	.001	5.39 to 25.00	8.06 ± 1.99	.0006	2.86 to 13.27

*Abbreviations:* CI, confidence interval; DBP, diastolic blood pressure; SBP, systolic blood pressure. Fourth quartile: warmest temperature; first quartile: coldest temperature.

## References

[1] Calkins H, Hindricks G, Cappato R (2017). 2017 HRS/EHRA/ECAS/APHRS/SOLAECE expert consensus statement on catheter and surgical ablation of AF. Heart Rhythm.

[2] Packer DL, Kowal RC, Wheelan KR (2013). Cryoballoon ablation of pulmonary veins for paroxysmal AF: first results of the North American Arctive Front (STOP AF) pivotal trial. J Am Coll Cardiol.

[3] Kuck KH, Brugada J, Fürnkranz A (2016). Cryoballoon or radiofrequency ablation for paroxysmal AF. N Engl J Med.

[4] Ketels S, Houben R, Van Beeumen K (2008). Incidence, timing, and characteristics of acute changes in heart rate during ongoing circumferential pulmonary vein isolation. Europace.

[5] Peyrol M, Barraud J, Koutbi L (2016). Vagal reactions during cryoballoon-based pulmonary vein isolation: a clue for autonomic nervous system modulation?. Biomed Res Int.

[6] Oswald H, Klein G, Koening T (2010). Cryoballoon pulmonary vein isolation temporarily modulates the intrinsic cardiac autonomic nervous system. J Interv Card Electrophysiol.

[7] Aytemir K, Gurses KM, Yalcin MU (2015). Safety and efficacy outcomes in patients undergoing pulmonary vein isolation with second-generation cryoballoon. Europace.

[8] Yorgun H, Aytemir K, Canpolat U (2014). Additional benefit of cryoballoon-based AF ablation beyond pulmonary vein isolation: modification of ganglionated plexi. Europace.

[9] Sun L, Dong JZ, DU X (2017). Prophylactic atropine administration prevents vasovagal response induced by cryoballoon ablation in patients with atrial fibrillation. Pacing Clin Electrophysiol.

[10] Kajiyama T, Miyazaki S, Watanabe T (2017). Circulatory dynamics during pulmonary vein isolation using the second-generation cryoballoon. J Am Heart Assoc.

[11] Zipes DP, Stevenson WG, Jalife J (2018). Cardiac Electrophysiology: From Cell to Bedside.

[12] Garabelli P, Stavrakis S, Kenney JFA, Po SS (2018). Effect of 28-mm cryoballoon ablation on major atrial ganglionated plexi. JACC Clin Electrophysiol.

[13] Pappone C, Santinelli V, Manguso F (2004). Pulmonary vein denervation enhances long-term benefit after circumferential ablation for paroxysmal AF. Circulation.

[14] Miyazaki S, Nakamura H, Taniguchi H (2016). Impact of the order of the targeted pulmonary vein on the VR during second-generation cryoballoon ablation. Heart Rhythm.

[15] Chebini A, Dilli E (2019). Cold stimulus headache. Curr Neurol Neurosci Rep.

[16] Foreman RD, Garrett KM, Blair RW (2015). Mechanisms of cardiac pain. Compr Physiol.

[17] Guckel D, Schmidt A, Gutleben K (2020). Pulmonary vein isolation and beyond: predictive value of vagal reactions in second-generation cryoballoon ablation for the outcome of persistent atrial fibrillation. Heart Rhythm.

[18] Hou Y, Scherlag BJ, Lin J (2007). Ganglionated plexi modulate extrinsic cardiac autonomic nerve input: effects on sinus rate, atrioventricular conduction, refractoriness, and inducibility of atrial fibrillation. J Am Coll Cardiol.

